# ARDS: hidden perils of an overburdened diagnosis

**DOI:** 10.1186/s13054-022-04271-y

**Published:** 2022-12-17

**Authors:** Martin J. Tobin

**Affiliations:** Division of Pulmonary and Critical Care Medicine, Hines Veterans Affairs Hospital, Loyola University of Chicago Stritch School of Medicine, Hines, IL 60141 USA

## Abstract

A diagnosis of ARDS serves as a pretext for several perilous clinical practices. Clinical trials demonstrated that tidal volume 12 ml/kg increases patient mortality, but 6 ml/kg has not proven superior to 11 ml/kg or anything in between. Present guidelines recommend 4 ml/kg, which foments severe air hunger, leading to prescription of hazardous (yet ineffective) sedatives, narcotics and paralytic agents. Inappropriate lowering of tidal volume also fosters double triggering, which promotes alveolar overdistention and lung injury. Successive panels have devoted considerable energy to developing a more precise definition of ARDS to homogenize the recruitment of patients into clinical trials. Each of three pillars of the prevailing Berlin definition is extremely flimsy and the source of confusion and unscientific practices. For doctors at the bedside, none of the revisions have enhanced patient care over that using the original 1967 description of Ashbaugh and colleagues. Bedside doctors are better advised to diagnose ARDS on the basis of pattern recognition and instead concentrate their vigilance on resolving the numerous hidden dangers that follow inevitably once a diagnosis has been made.

Critical care is more closely entwined with the acute respiratory distress syndrome (ARDS) than with any other diagnosis. In 2017, several journals showcased articles celebrating the 50-year anniversary of Ashbaugh and coauthors’ original article [1]. Ruminating on why an inordinately large number of journal articles and sessions at critical care congresses are devoted to a syndrome that accounts for less than one ICU admission every two months (according to strict definition criteria), Gattinoni surmised that the primary reason was sentimentality [2].

Most intensivists—though not all [3]—feel indebted to Ashbaugh and colleagues for describing a previously unrecognized syndrome in 1967. After an interval of 40 years, two landmark clinical trials demonstrated that ventilator tidal volume was a decisive determinant of clinical outcome: patients receiving 12 ml/kg exhibited a 22.1% higher mortality than patients ventilated with 6 ml/kg [4, 5]. In reality, bedside doctors had already turned away from higher tidal volumes before publication of these trials [6–8]. Avoiding tidal volume 12 ml/kg remains the sole therapeutic step proven to decrease ARDS mortality. Given that tidal volume 12 ml/kg is not used in any ventilated patient, making a diagnosis of ARDS has no impact on bedside decisions [3, 9].

A diagnosis of ARDS serves as a pretext for several perilous practices (Table 1). Following publication of the positive trials, ARDS guidelines promoted the use of tidal volume 6 ml/kg, although 6 ml/kg has never been shown to be superior to 11 ml/kg or anything in between. The most recent guidelines are more extreme: recommending 4 ml/kg, which entails an unnatural tidal volume of 280 ml for an average person [10]. Critically ill patients have inflamed lungs and stimulation of sensory receptors produces heightened respiratory drive and dyspnea [11]. Dyspneic patients react by attempting deeper inspirations. When a low tidal volume setting impedes this response, agonizing dyspnea is ensured through corollary discharge from the medulla oblongata to the cerebral cortex [12]. Dyspnea is amplified by hypercapnia that is axiomatic to hypoventilation; a rise in PaCO_2_ from 45 to 50 mmHg induces increases in minute ventilation of 25 L/min and tremendous air hunger [13].

**Table 1:** Perils that ensue upon making a diagnosis of ARDS

Protocol mandate	Physiologic consequences	Clinical problems
Prescription of tidal volume 6 ml/kg in all patients, irrespective of plateau pressure	Severe air hunger ensues when delivered tidal volume does not match heightened stimulation of sensory receptors	Sedatives, opiates, and paralytic agents do not allay air hunger but contribute to complications
	If mechanical inspiratory time is shorter than neural inspiratory time, double triggering is inevitable	Despite adjusting a ventilator to deliver 6 ml/kg, the patient actually receives 12 ml/kg
Fixed PEEP options	Constraints imposed by use of PEEP-F_I_O_2_ table	If F_I_O_2_ is 0.60: patient got either PEEP 10 or 20 cm H_2_O with no other options If F_I_O_2_ is 0.80: patient got either PEEP 14 or 22 cm H_2_O with no other options

The only physiological variable that discriminated between the two positive clinical trials [4, 5] and the three negative trials [14–16] was average airway plateau pressure. Patients with plateau pressures greater than 32 cm H_2_O had significantly higher mortality [17]. Plateau pressure is the variable that best reflects alveolar overdistention and likelihood of lung injury. Instead of pivoting on plateau pressure, guideline panelists presented recommendations in terms of tidal volume expressed as milliliters per kilogram. This is analogous to managing a hypertensive emergency by titrating dosage of antihypertensive agents according to patient body weight rather than adjusting dosage in response to iterative changes in blood pressure. The most recent re-analysis of data from the five trials of high versus low tidal volume have finally come around to a conclusion that tidal volume should no longer be ordered in terms of milliliters per kilogram [18]. The unthinking recourse to 6 ml/kg, perhaps the most omnipresent order of ICU residents, has finally been sanctioned as scientifically flawed [18, 19].

When receiving unnaturally low tidal volumes, patients rebel against torturous air hunger and buck the ventilator [20]. Caregivers use sedative, narcotic and paralyzing agents to combat recalcitrant patients and restrain them on the Procrustean bed of 6 ml/kg. Sedative and narcotic agents do not allay air hunger [21, 22], and neuromuscular blockers aggravate dyspnea by removing behavioral clues that alert caregivers to patient discomfort [12]. For clinicians who have cared for ventilated patients over the past 40 years, it is disheartening to observe large doses of sedatives, narcotics and paralyzing agents being prescribed nonchalantly, reversing the great strides in the 1980s–1990s to curtail their use. It contravenes every principle of physiology to prescribe unnaturally low tidal volumes in patients with plateau pressures in the low 20 s.

The ARDS-Network web resource and guidelines promote a one-size-fits-all approach to mechanical ventilation. Protocol advocates, ungrounded in physiology, do not recognize that low tidal volume is necessarily accompanied by shortening of mechanical inspiratory time [11]. Once mechanical inspiratory time becomes less than neural inspiratory time, double triggering is inevitable. Protocol enthusiasts believe they are delivering a tidal volume of 6 ml/kg, but the patient is actually receiving 12 ml/kg—a setting proven to increase mortality [4, 5].

Managing patients according to the PEEP-F_I_O_2_ table of the ARDS-Network contradicts all principles of physiology and even common sense. If F_I_O_2_ was set at 60%—a common choice in ARDS – the patient got either PEEP 10 or 20 cm H_2_O, with no other options [23]. If F_I_O_2_ was set at 80%, the patient got either PEEP 14 or 22 cm H_2_O, with no other options.

Definitions of ARDS have been revised several times since Ashbaugh’s original description. As each new formulation was unfurled, authors justified their revision by specifying grave flaws in the antecedent definition and promising that emendations will remedy past blemishes. Not long after Ashbaugh and colleagues heralded the new syndrome, Dr. Murray became a vociferous critic, counseling clinicians against making the diagnosis [24]. Dr. Murray made a subsequent volte-face, recommending that the diagnosis was best made using a lung injury score [25]. Six years later, the American-European Consensus Committee claimed that weaknesses in the Murray score merited a new definition [26]. In 2012, the Berlin Task Force listed numerous flaws in its predecessor and announced that their definition was the first attempt to link an international consensus panel endorsed by professional societies with an empirical evaluation of the revised criteria in thousands of patients [27]. In recent weeks, intimations have appeared that another iteration is on its way [28]. Doing the same thing over and over and expecting different results is something on which Einstein commented. His conclusion was not flattering.

I recently pointed out that a fetish fixation on the Berlin definition of ARDS may have contributed to patient mortality at the height of the COVID-19 pandemic [29]. Some members of the Berlin Task Force took umbrage at this inference [30]. The Task Force, however, could not have foreseen how their definition was to be employed during a subsequent pandemic. The WHO guidelines on COVID-19 [31] made a clear link between the diagnosis of ARDS (their citation #17 specifies the Berlin definition) and encouraging early endotracheal intubation, which was subsequently shown to contribute to increased Covid mortality [32]. WHO stated explicitly that “Hypoxemic respiratory failure in ARDS … usually requires mechanical ventilation” (context conveyed the invasive form). This is not true. Many patients with ARDS are sustained with noninvasive ventilation or supplemental oxygen [33, 34]. A PubMed search will reveal numerous authors forging links between making a diagnosis of ARDS in Covid patients and early intubation; see, for example, the report by Ziehr et al. [35] (their citation #7 specifies the Berlin definition), upon which Yaroshetskiy et al. [36] subsequently commented.

The definition put forward by Ashbaugh and colleagues consisted of simple qualitative descriptors (severe dyspnea, tachypnea, hypoxemia, decreased lung compliance, alveolar infiltrates). Authors of subsequent definitions have acted as if they subscribed to Lord Kelvin’s dictum on numerical precision.^1^ In reality, it is the numerical encasing of the three pillars (of the Berlin definition) that render them very rickety (Fig. 1). The criteria for radiographic infiltrates achieve dismal interrater agreement, with a kappa score of 0.296 [37].

**Fig. 1:**
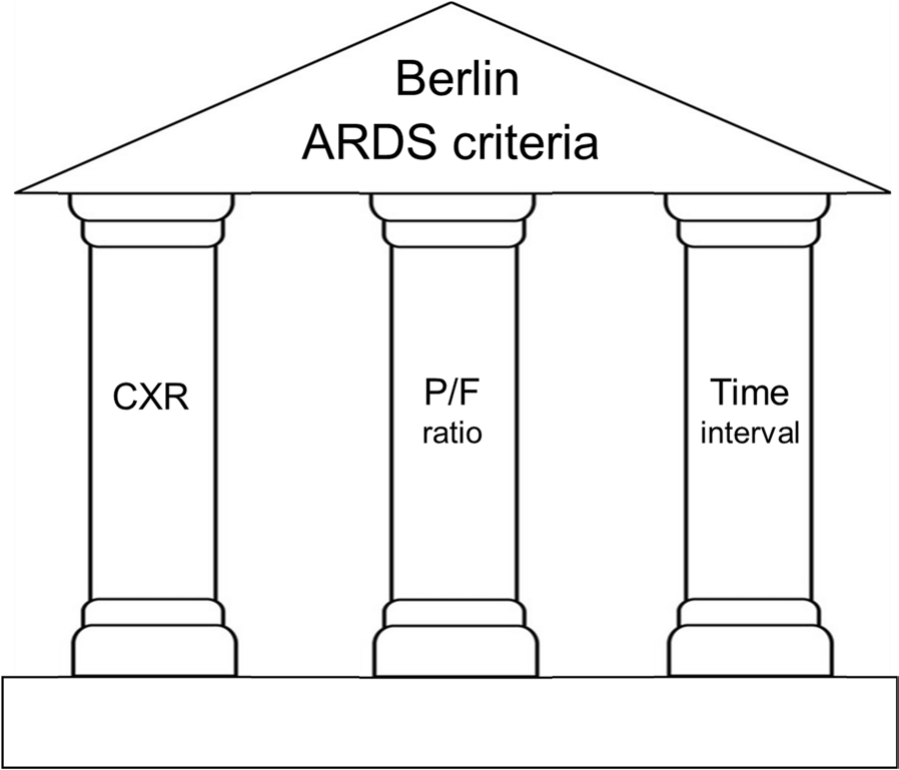
The Berlin criteria for the definition of ARDS consist of three pillars, each of which is flimsy. Chest X-ray (CXR) infiltrates have a kappa interrater agreement score of 0.296. Arterial PO_2_ to fractional inspired oxygen (P/F ratio), an index of patient oxygenation, is physiologically flawed and not fit for purpose. A 7-day interval between the inciting insult and onset of symptoms is whimsical

The Berlin group specifies that ARDS can be diagnosed legitimately only if respiratory failure is identified within 7 days of a recognized insult. This whimsical time limit was the source of considerable confusion during the Covid pandemic, with authors believing that Covid pneumonia did not represent ARDS because respiratory failure occurred 8–12 days after first symptoms [38].

Severe hypoxemia has always featured as a constitutive prerequisite for ARDS diagnosis. Definitions subsequent to Ashbaugh consistently express hypoxemia in terms of PaO_2_/F_I_O_2_ ratio. Murray and colleagues selected the ratio as an exemplar of abnormal gas exchange because it “is more easily calculated from information routinely available in patients’ charts” [25]. Seldom did an intention of not wanting to burden others backfire so spectacularly. It was already known that PaO_2_ has a curvilinear relationship with F_I_O_2_, which varies with the degree of ventilation-perfusion inequality and shunt [39, 40]. In patients with ARDS and a fixed shunt, alterations in F_I_O_2_ cause PaO_2_/F_I_O_2_ to fluctuate unpredictably by greater than 100 mmHg [41]. In a group of patients who fulfilled all ARDS criteria, administration of 100% oxygen for 30 min produced an increase in PaO_2_/F_I_O_2_ to such an extent that 58.5% of the patients no longer met ARDS criteria [42].

PaO_2_ is one of the most precise measurements across medicine. Several organs, such as the carotid bodies, respond to miniscule changes in PaO_2_ and it is a key determinant of oxygen delivery to the brain and heart. In contrast, PaO_2_/F_I_O_2_ plays no role in any biological process. PaO_2_, not PaO_2_/F_I_O_2_ or oxygen saturation (SaO_2_), was the decisive clue in solving the mystery of why some Covid patients exhibited silent (happy) hypoxia [43].

Galvanized by the invariable inclusion of PaO_2_/F_I_O_2_ in successive ARDS definitions, thousands of authors have reported patient oxygenation in terms of this ratio. In an early Covid series, authors from Seattle, one of the cradles of critical care, reported oxygenation solely in terms of PaO_2_/F_I_O_2_ with no mention of PaO_2_ [44]. PaO_2_/F_I_O_2_ ratio is perhaps the most glaring example of Gresham’s law in medicine, where a bad measurement drives out a good measurement.

It is cautionary for intensivists to realize that a diagnosis considered iconic of critical care [2] is defined by the most unscientific of criteria. It is understandable that researchers would wish to refine recruitment criteria to homogenize the entry of patients into clinical trials, but this housekeeping chore could be better handled through private communications among trialists without distracting bedside doctors from more momentous matters. Patients would be better served by clinicians concentrating their attention on physiological problems unique to each individual patient and developing customized solutions [45].

When I work as a bedside doctor, I consider the diagnosis of ARDS to be a useful, if somewhat ragbag, label. Like many syndromes, ARDS is crude and lacks precise defining boundaries of clinical disorders such as Legionnaires disease or hemiplegia consequent to internal-capsule hemorrhage. I reach a diagnosis of ARDS based on tacit knowledge and recognition of a constellation of dyspnea, physical signs of respiratory effort [45], hypoxemia, and radiographic infiltrates without getting pedantic about numbers or finicky about distribution patterns [46]. Making a diagnosis of ARDS is not a final terminus and I carry on searching for the underlying cause: treatment of pneumococcal pneumonia differs from that of pancreatitis.

ARDS is overburdened by unrealistic aspirations of researchers (trialists), hoping to employ sociological stratagems to transform an ineffable entity into an ontological thing of nature (a “natural kind”) [28, 29]. Science evolves differently than a Hans Christian Andersen fairytale. It is time for researchers to stop yearning after a glorious swan and accept ARDS as something of an ugly duckling. If successive panels of leading pulmonary and critical care experts cannot come up with a scientifically satisfying definition of ARDS, is it really likely that patient representatives (a recent proposal [28] will resolve the deep epistemological and ontological conundrums at its core? Nobelist Peter Medawar, foremost epistemologist of biology of the last century, warned of the danger of venerating definitions, and their tendency to constrain the mind rather than to liberate it [47]. Labels have no more than a nominalist usage, and craving after immutable apodictic certainty is perilous.

## Data Availability

When writing the manuscript, I did not have access to any special sets of data beyond what is available in regularly published articles. As such I cannot provide any special access to data sets that readers might desire/

## Footnotes

1 “When you can measure what you are speaking about, and express it in numbers, you know something about it; but when you cannot measure it, when you cannot express it in numbers, your knowledge is of a meagre and unsatisfactory kind: it may be the beginning of knowledge, but you have scarcely, in your thoughts, advanced to the stage of *science*, whatever the matter may be.” (delivered 3 May 1883).

## References

[1] Ashbaugh DG, Bigelow DB, Petty TL, Levine BE (1967). Acute respiratory distress in adults. Lancet.

[2] Gattinoni L, Quintel M (2016). Fifty years of research in ARDS: why is acute respiratory distress syndrome so important for critical care?. Am J Respir Crit Care Med.

[3] Vincent JL, Santacruz C (2016). Do we need ARDS?. Intensive Care Med.

[4] Amato MB, Barbas CS, Medeiros DM, Magaldi RB, Schettino GP, Lorenzi-Filho G, Kairalla RA, Deheinzelin D, Munoz C, Oliveira R, Takagaki TY, Carvalho CR (1998). Effect of a protective-ventilation strategy on mortality in the acute respiratory distress syndrome. N Engl J Med.

[5] Acute Respiratory Distress Syndrome Network, Brower RG, Matthay MA, Morris A, Schoenfeld D, Thompson BT, Wheeler A. Ventilation with lower tidal volumes as compared with traditional tidal volumes for acute lung injury and the acute respiratory distress syndrome. N Engl J Med. 2000;342(18):1301–8. 10.1056/NEJM200005043421801 10793162

[6] Carmichael LC, Dorinsky PM, Higgins SB, Bernard GR, Dupont WD, Swindell B, Wheeler AP (1996). Diagnosis and therapy of acute respiratory distress syndrome in adults: an international survey. J Crit Care.

[7] Hickling KG, Henderson SJ, Jackson R (1990). Low mortality associated with low volume pressure limited ventilation with permissive hypercapnia in severe adult respiratory distress syndrome. Intensive Care Med.

[8] Deans KJ, Minneci PC, Danner RL, Eichacker PQ, Natanson C (2010). Practice misalignments in randomized controlled trials: Identification, impact, and potential solutions. Anesth Analg.

[9] Tobin MJ (2020). Does making a diagnosis of ARDS in patients with coronavirus disease 2019 matter?. Chest.

[10] Fan E, Del Sorbo L, Goligher EC, Hodgson CL, Munshi L, Walkey AJ, Adhikari NKJ, Amato MBP, Branson R, Brower RG, Ferguson ND, Gajic O, Gattinoni L, Hess D, Mancebo J, Meade MO, McAuley DF, Pesenti A, Ranieri VM, Rubenfeld GD, Rubin E, Seckel M, Slutsky AS, Talmor D, Thompson BT, Wunsch H, Uleryk E, Brozek J, Brochard LJ; American Thoracic Society, European Society of Intensive Care Medicine, and Society of Critical Care Medicine. An Official American Thoracic Society/European Society of Intensive Care Medicine/Society of Critical Care Medicine Clinical Practice Guideline: Mechanical ventilation in adult patients with acute respiratory distress syndrome. Am J Respir Crit Care Med. 2017;195(9):1253–63. 10.1164/rccm.201703-0548ST 28459336

[11] Tobin MJ, Laghi F, Jubran A (2012). Ventilatory failure, ventilator support and ventilator weaning. Compr Physiol.

[12] Banzett RB, Similowski T, Brown R, Tobin MJ (2012). Addressing respiratory discomfort in the ventilated patient. Principles and practice of mechanical ventilation.

[13] Tobin MJ, Gardner WN, Tobin MJ (1998). Monitoring of the control of ventilation. Principles and practice of intensive care monitoring.

[14] Stewart TE, Meade MO, Cook DJ, Granton JT, Hodder RV, Lapinsky SE, Mazer CD, McLean RF, Rogovein TS, Schouten BD, Todd TR, Slutsky AS. Evaluation of a ventilation strategy to prevent barotrauma in patients at high risk for acute respiratory distress syndrome. Pressure- and Volume-Limited Ventilation Strategy Group. N Engl J Med. 1998;338(6):355–61. 10.1056/NEJM199802053380603 9449728

[15] Brochard L, Roudot-Thoraval F, Roupie E, Delclaux C, Chastre J, Fernandez-Mondéjar E, Clémenti E, Mancebo J, Factor P, Matamis D, Ranieri M, Blanch L, Rodi G, Mentec H, Dreyfuss D, Ferrer M, Brun-Buisson C, Tobin M, Lemaire F. Tidal volume reduction for prevention of ventilator-induced lung injury in acute respiratory distress syndrome. The Multicenter Trail Group on Tidal Volume reduction in ARDS. Am J Respir Crit Care Med. 1998;158(6):1831–8. 10.1164/ajrccm.158.6.9801044 9847275

[16] Brower RG, Shanholtz CB, Fessler HE, Shade DM, White P, Wiener CM, Teeter JG, Dodd-o JM, Almog Y, Piantadosi S (1999). Prospective, randomized, controlled clinical trial comparing traditional versus reduced tidal volume ventilation in acute respiratory distress syndrome patients. Crit Care Med.

[17] Tobin MJ (2000). Culmination of an era in research on the acute respiratory distress syndrome. N Engl J Med.

[18] Goligher EC, Costa ELV, Yarnell CJ, Brochard LJ, Stewart TE, Tomlinson G, Brower RG, Slutsky AS, Amato MPB (2021). Effect of lowering Vt on mortality in acute respiratory distress syndrome varies with respiratory system elastance. Am J Respir Crit Care Med.

[19] Tobin MJ (2021). The dethroning of 6 ml/kg as the "go-to" setting in acute respiratory distress syndrome. Am J Respir Crit Care Med.

[20] Tobin MJ, Jubran A, Laghi F, Tobin MJ (2012). Fighting the ventilator. Principles and practice of mechanical ventilation.

[21] Simon ST, Higginson IJ, Booth S, Harding R, Weingärtner V, Bausewein C. Benzodiazepines for the relief of breathlessness in advanced malignant and non-malignant diseases in adults. Cochrane Database Syst Rev. 2016;10(10) 10.1002/14651858.CD007354.pub3 PMC6464146 27764523

[22] Ekström M, Ferreira D, Chang S, Louw S, Johnson MJ, Eckert DJ, Fazekas B, Clark KJ, Agar MR, Currow DC; Australian National Palliative Care Clinical Studies Collaborative. Effect of regular, low-dose, extended-release morphine on chronic breathlessness in chronic obstructive pulmonary disease: The BEAMS randomized clinical trial. JAMA. 2022;328(20):2022–32. 10.1001/jama.2022.20206 PMC9682426 36413230

[23] Brower RG, Lanken PN, MacIntyre N, Matthay MA, Morris A, Ancukiewicz M, Schoenfeld D, Thompson BT; National Heart, Lung, and Blood Institute ARDS Clinical Trials Network. Higher versus lower positive end-expiratory pressures in patients with the acute respiratory distress syndrome. N Engl J Med. 2004;351(4):327–36. 10.1056/NEJMoa032193 15269312

[24] Murray JF (1975). Editorial: The adult respiratory distress syndrome (may it rest in peace). Am Rev Respir Dis.

[25] Murray JF, Matthay MA, Luce JM, Flick MR (1988). An expanded definition of the adult respiratory distress syndrome. Am Rev Respir Dis.

[26] Bernard GR, Artigas A, Brigham KL, Carlet J, Falke K, Hudson L, Lamy M, Legall JR, Morris A, Spragg R. The American-European Consensus Conference on ARDS. Definitions, mechanisms, relevant outcomes, and clinical trial coordination. Am J Respir Crit Care Med. 1994;149(3 Pt 1):818–24. 10.1164/ajrccm.149.3.7509706 7509706

[27] ARDS Definition Task Force, Ranieri VM, Rubenfeld GD, Thompson BT, Ferguson ND, Caldwell E, Fan E, Camporota L, Slutsky AS. Acute respiratory distress syndrome: the Berlin Definition. JAMA. 2012;307(23):2526–33. 10.1001/jama.2012.5669 22797452

[28] Ranieri VM, Rubenfeld G, Slutsky AS. Rethinking ARDS After COVID-19. If a "better" definition is the answer, what is the question? Am J Respir Crit Care Med. 2022. doi: 10.1164/rccm.202206-1048CP. 10.1164/rccm.202206-1048CP PMC9896638 36150099

[29] Tobin MJ (2022). Defining ARDS (again): a plea for honesty. Am J Respir Crit Care Med.

[30] Ranieri VM, Rubenfeld G, Slutsky AS (2022). Reply to: Defining ARDS (again): a plea for honesty. Am J Respir Crit Care Med.

[31] World Health Organization. Clinical management of severe acute respiratory infection when novel coronavirus (2019-nCoV) infection is suspected: Interim guidance 28 January 2020 https://apps.who.int/iris/bitstream/handle/10665/330893/WHO-nCoV-Clinical-2020.3-eng.pdf?sequence=1&isAllowed=y. Date last accessed: 3 Dec 2022

[32] Doidge JC, Gould DW, Ferrando-Vivas P, Mouncey PR, Thomas K, Shankar-Hari M, Harrison DA, Rowan KM (2021). Trends in intensive care for patients with COVID-19 in England, Wales, and Northern Ireland. Am J Respir Crit Care Med.

[33] Antonelli M, Conti G, Esquinas A, Montini L, Maggiore SM, Bello G, Rocco M, Maviglia R, Pennisi MA, Gonzalez-Diaz G, Meduri GU (2007). A multiple-center survey on the use in clinical practice of noninvasive ventilation as a first-line intervention for acute respiratory distress syndrome. Crit Care Med.

[34] Messika J, Ben Ahmed K, Gaudry S, Miguel-Montanes R, Rafat C, Sztrymf B, Dreyfuss D, Ricard JD (2015). Use of high-flow nasal cannula oxygen therapy in subjects with ARDS: a 1-year observational study. Respir Care.

[35] Ziehr DR, Alladina J, Petri CR, Maley JH, Moskowitz A, Medoff BD, Hibbert KA, Thompson BT, Hardin CC (2020). Respiratory pathophysiology of mechanically ventilated patients with COVID-19: a cohort study. Am J Respir Crit Care Med.

[36] Yaroshetskiy AI, Avdeev SN, Konanykhin VD (2020). Acute respiratory distress syndrome in COVID-19: do all these patients definitely require intubation and mechanical ventilation?. Am J Respir Crit Care Med.

[37] Goddard SL, Rubenfeld GD, Manoharan V, Dev SP, Laffey J, Bellani G, Pham T, Fan E (2018). The Randomized Educational Acute Respiratory Distress Syndrome Diagnosis Study: a trial to improve the radiographic diagnosis of acute respiratory distress syndrome. Crit Care Med.

[38] Li X, Ma X (2020). Acute respiratory failure in COVID-19: is it "typical" ARDS?. Crit Care.

[39] West JB (1977). State of the art: ventilation-perfusion relationships. Am Rev Respir Dis.

[40] Dantzker DR (1982). Gas exchange in the adult respiratory distress syndrome. Clin Chest Med.

[41] Gowda MS, Klocke RA (1997). Variability of indices of hypoxemia in adult respiratory distress syndrome. Crit Care Med.

[42] Ferguson ND, Kacmarek RM, Chiche JD, Singh JM, Hallett DC, Mehta S, Stewart TE (2004). Screening of ARDS patients using standardized ventilator settings: influence on enrollment in a clinical trial. Intensive Care Med.

[43] Tobin MJ, Laghi F, Jubran A (2020). Why COVID-19 silent hypoxemia is baffling to physicians. Am J Respir Crit Care Med.

[44] Bhatraju PK, Ghassemieh BJ, Nichols M, Kim R, Jerome KR, Nalla AK, Greninger AL, Pipavath S, Wurfel MM, Evans L, Kritek PA, West TE, Luks A, Gerbino A, Dale CR, Goldman JD, O'Mahony S, Mikacenic C (2020). Covid-19 in critically ill patients in the Seattle region: case series. N Engl J Med.

[45] Tobin MJ (2019). Why physiology is critical to the practice of medicine: a 40-year personal perspective. Clin Chest Med.

[46] Aberle DR, Wiener-Kronish JP, Webb WR, Matthay MA (1988). Hydrostatic versus increased permeability pulmonary edema: diagnosis based on radiographic criteria in critically ill patients. Radiology.

[47] Medawar P, Medawar J. Aristotle to zoos: a philosophical dictionary of biology. Oxford University Press. Oxford, 1983, p. 66.

